# Supplementary figures and images for: Management of possible serious bacterial infection in young infants where referral is not possible in the context of existing health system structure in Ibadan, South-west Nigeria
Source: PLoS One. 2021 Mar 30;16(3):e0248720. doi: 10.1371/journal.pone.0248720 (PMC8009401; doi:10.1371/journal.pone.0248720)

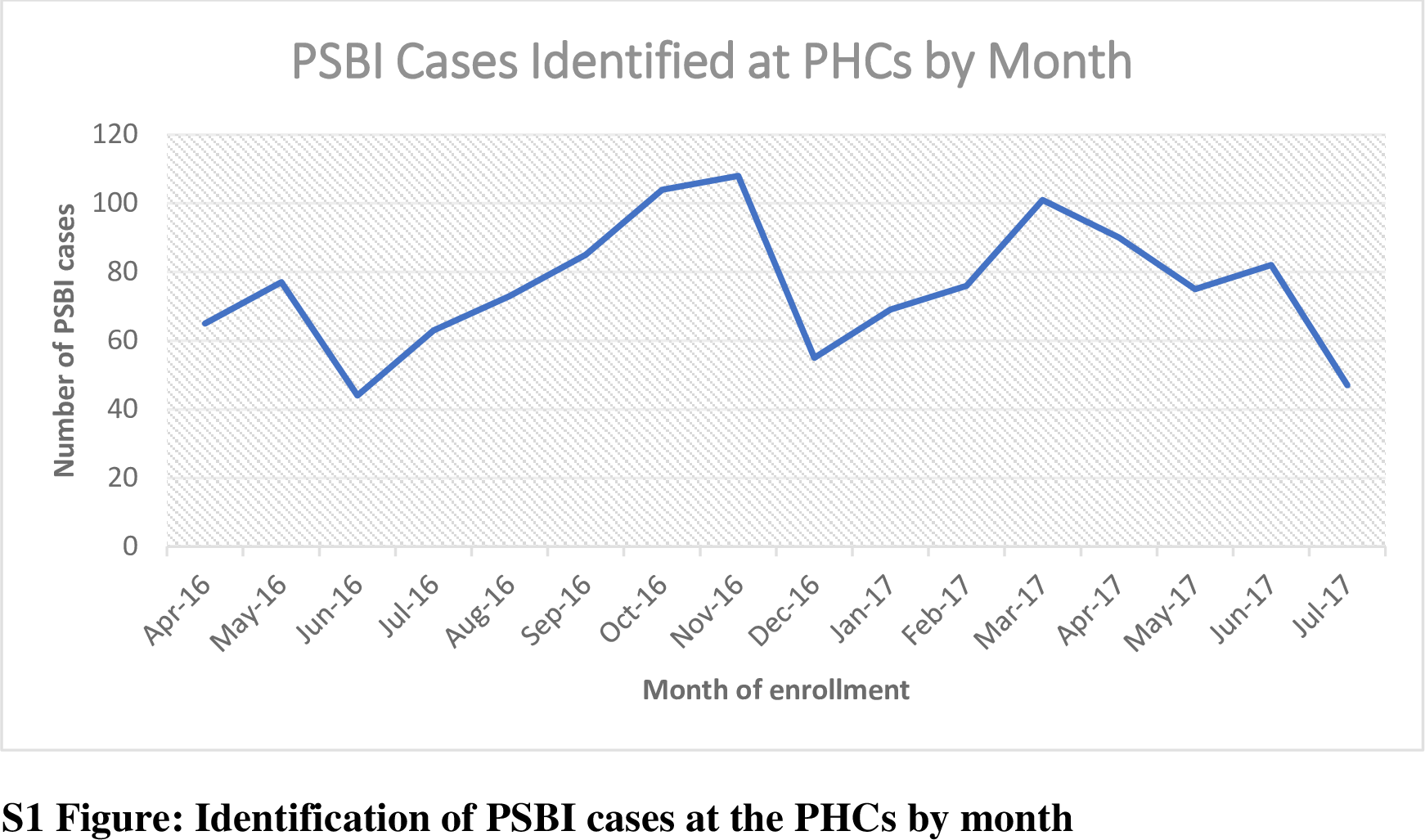

Supplement: S1 Fig — (TIF) [file pone.0248720.s001.tif]
