# Supplementary material for: Management of possible serious bacterial infection in young infants where referral is not possible in the context of existing health system structure in Ibadan, South-west Nigeria
Source: PLoS One. 2021 Mar 30;16(3):e0248720. doi: 10.1371/journal.pone.0248720 (PMC8009401; doi:10.1371/journal.pone.0248720)
Supplement: S1 Table — (PDF) [file pone.0248720.s002.pdf]

S1 Table. Staff category in each health facility

Health Facility	Category of Leader	Nurses	CHOs	CHEWs	HAs	Voluntary HWs	CORPs
Lalupon	CNO	1	0	4	3	2	2
Ejioku	CNO	1	2	4	6	1	2
Monatan	SNO	2	0	3	6	1	2
Iyana Church	CNO	2	0	3	6	1	2
Olorunda	CNO	2	1	2	2	1	2
Alegongo	CHEW	0	1	6	4	1	2
Iyana Ore	CHEW	0	0	1	2	0	1
Oyediji	CHEW	0	1	0	1	0	1

CNO: Chief Nursing Officer; SNO: Senior Nursing Officer; CHEW: Community Health Extension Worker; HA: Health Assistant; HW: Health Worker; CORPs: Community Resource Persons
