# Supplementary material for: Management of possible serious bacterial infection in young infants where referral is not possible in the context of existing health system structure in Ibadan, South-west Nigeria
Source: PLoS One. 2021 Mar 30;16(3):e0248720. doi: 10.1371/journal.pone.0248720 (PMC8009401; doi:10.1371/journal.pone.0248720)
Supplement: S1 File — (PDF) [file pone.0248720.s003.pdf]

FIELD SUPERVISION CHECKLIST

PSBI Implementation Study

CHEW.

Instruction:

This checklist should be administered on a CORPS a minimum of two times per month.

CORPs' Name_________________________________ CORPs' ID_______ Date of Supervision__________

Visit 1st 2nd

Home Visit for Pregnant Woman

Number of Pregnant women identified	________		Comment
All item on Form 1 entered correctly	yes	No	
Follow up calendar for each pregnant woman identified is correct	Yes	No	

Review assessment of two pregnant women

Pregnant women 1:

Name ____ Serial No. on Form 1. ______ Age ________

Check Comment

- CORP actually visited the pregnant woman yes No
- CORP actually interacted with the pregnant woman yes No
- Pregnant woman has her mother and baby card yes No

Pregnant women 2:

Name _________________________________Serial No. on Form 1. ____ Age_____

Check

- CORP actually visited the pregnant woman yes No
- CORP actually interacted with the pregnant woman yes No
- Pregnant woman has her mother and baby card yes No

Home Visit for Newborn

All items on Form 2 correctly entered	Yes	No
Number of Newborns identified	_________	
Calendar of home visit on each newborn is correct	Yes	No

All danger signs assessed for each newborn Yes No

Number of newborns with a danger sign _________

All newborns with a danger sign assessed by the study/facility Nurse Yes No

If a newborn with a danger sign is not assessed by the study /facility Nurse, give reason
____

____

Review two new-borns

Newborn 1.

Name _______________________________ Infant ID _____ Age ___ Sex: M F

Check: Comment

- CORP actually visited the newborn at home Yes No
- Newborn visited within 24hours of birth Yes No
- Number of visits indicated by CORP agrees with mother's account Yes No
- CORP actually examined the newborn Yes No
- If newborn has a danger sign, a Nurse actually examined the newborn Yes No

Newborn 2.

Name _______________________________ Infant ID _____ Age ____Sex: M F

Check:

- CORP actually visited the newborn at home Yes No
- Newborn visited within 24hours of birth Yes No
- Number of visits indicated by CORP agrees with mother's account Yes No
- CORP actually examined the newborn Yes No
- If newborn has a danger sign, a Nurse actually examined the newborn Yes No

JOB AIDS

- Counseling cards present and in good condition Yes No
- Weighing scale present and function correctly Yes No
- Thermometer present and function correctly Yes No
- Timer present and function correctly Yes No
- Mother and baby cards available Yes No
- Form 1 available yes No
- Form 2 available Yes No
- Consent home for home visit available yes No

Areas of weakness

____

____

Areas of emphasis during next visit

Field Supervisor Name ___________________________signed ____________ Date ______

FIELD SUPERVISION CHECKLIST

PSBI Implementation Study

TREATMENT NURSE.

Instruction:

This checklist should be administered on a Treatment Nurse.

Nurse Name______________________________Nurse ID_______ Date of Supervision__________

Visit 1st 2nd

FORM3: Comment

All items on Form 3 correctly entered Yes No

Classification for possible serious bacterial infection on all form 3 correct Yes No

All other classifications are correct Yes No

All newborns with pink classification correctly referred yes No

All referrals took place within 2 hours Yes No

All referrals took place to the correct reference health facility (UCH, ONI MEMORIAL,ADEOYO) Yes No

FORM 5:

All items on Form 5 correctly entered Yes No

The drug dosage correctly calculated Yes No

The drugs correctly administered according to protocol Yes No

Signs of critical illness, serious illness and fast breathing correct assessed Yes No

GENERAL:

All non-treated Non Hospitalized are followed up in two weeks Yes No

All non-treated Hospitalized are followed at home in two weeks Yes No

JOB AIDS

▪ Weighing scale present and function correctly	Yes	No
▪ Thermometer present and function correctly	Yes	No
▪ Timer present and function correctly	Yes	No
▪ Form 3 available	Yes	No
▪ Form 4 available	Yes	No
▪ Form 5 available	Yes	No
▪ Consent form for inclusion into study	yes	No

Field Supervisor Name ___________________________signed________________ Date ______
