# Supplementary material for: Management of possible serious bacterial infection in young infants where referral is not possible in the context of existing health system structure in Ibadan, South-west Nigeria
Source: PLoS One. 2021 Mar 30;16(3):e0248720. doi: 10.1371/journal.pone.0248720 (PMC8009401; doi:10.1371/journal.pone.0248720)
Supplement: Attachment — Submitted filename: RESPONSE TO REVIEWERS .docx [file pone.0248720.s005.docx]

**REBUTTAL LETTER**

**Management of possible serious bacterial infection in young infants where referral is not possible in the context of existing health system structure in Ibadan, South-west Nigeria**

We appreciate the Editor for his useful suggestions. We have addressed all the suggestions in our current version and we believe the manuscript has been improved. In this letter, our responses are tabulated as in the third column. We have also included a copy of our manuscript with track changes.

The two Reviewers are also greatly appreciated for their comments.

Below are the responses to all the comments**:**

| **Reviewer** | **Comment** | **Response** |
| --- | --- | --- |
| Reviewer 1 | First, the Technical Support Unit was established for the research project, as explained (page 11). Was the TSU set up to continue after the project ended or did these end with the project? | The Technical Support Unit was set up purposely for the project as part of their role was to build capacity and support implementation of the project as stated in Panel 3. Line 214. |
|  | Second, the TSU included project supervisors and project nurses, which one assumes were hired for +the project itself. Were these new positions funded by the State MOH with budget lines for these positions to be retained at the end of the projects or were these positions funded by the projects and then positions terminated at the end of the study? | The TSU were funded by the project and their funding ended with the project. Since they were part of the University faculty and Hospital Staff, they were still available to offer technical support and share experiences for scale up to other states. Lines 204-213. |
|  | Third, the authors note the stipends for the CORPS as a limitation. The assumption is that these stipends ended after the project concluded and the CORPS no longer undertook these responsibilities. More details are required on this point as well. | The stipends ended with the project. More details are provided in Lines 508-510. |
|  | Four, you established a separate monitoring and supervision system, which would not be feasible to implement in the current system. | Part of the technical role of TSU is to demonstrate, supervise and monitor the implementation activities. Ideally monitoring and supervision should be part of PHC activities. However, this may not be feasible due to inadequate staff and finance. |
|  | Finally, the commodities were provided by the project, as noted in the limitations section. However, did families still have to pay for these (as they did for other medicines at the PHCs)? | No, the families did not pay for these commodities as they were provided by the project. This has been further clarified in logistics and commodities section lines 264-267 and also stated under limitation in lines 507-508 |
|  | Also why were there stockouts when the project was providing these (Table 3)? The authors should reassess their framing that this intervention was implemented through the existing system given it does not seem sustainable beyond the life of the project. | The state and the LGA did not provide drugs and other commodities (lines 265-266) which was identified as a barrier thus the need for discussing ways of preventing stockouts of commodities as stated in lines 488 - 491 |
|  | The data collection process is unclear. In the limitation section, you note the intensive monitoring and supervision undertaken (which would be difficult to replicate). The methods section provides an explanation of the oversight process including the checklist used in supplementary files. But it is not clear as to who was supervising the data collection (this information is not specified on page 14). | The data collection was supervised by the study supervisors as stated on lines 289-295 under monitoring, supervision, skills maintenance and process evaluation. Data collection was done by CORPs (Pregnancy register and postnatal visit CRFs), Health Facility Workers and study nurses (Assessment, classification and treatment form) in lines 291– 296. |
|  | Also more information is needed on how the case data was collected (e.g. births, identification of suspected cases, treatment adherence, follow up) – who collected it, how was it captured, etc… The assumption is that the CORPS collected this data using the CRFs, but that is never stated or explained. | This has been explained in lines 274– 276 (pregnancy and birth data were collected by CORPs and identification of sick infant and referral to PHC was also done by CORPs). Treatment was done by PHCs nurses and assisted by study nurses. Data on treatment adherence and follow-up were collected by and HFW and study nurses. Day 14 treatment outcome was done by study nurses, Lines 291-296. |
|  | The details provided on data collected and analysis of the implementation factors (barriers and facilitating) is weak and needs further explanation. Who collected the data? Were standard tools developed and used for observations, interactions and events? The fact that there were no formal interviews or focus groups is a limitation. The subjectivity of these findings also needs to be noted as a limitation in the discussion section. | Standardized supervisory checklist was used as stated in supplementary file 1. The barriers and enabling factors were identified during monitoring and evaluation of implementation process by direct observation and interaction with HFW as stated in lines 290 - 291  The qualitative component of the study comprised of In-depth interviews & Key Informant interview as well as focus group discussion were carried out which would be published as a separate manuscript. This has been included in lines 298 - 301. |
|  | The methods section was hard to follow. The sister study paper on Zaria, Wammanda et al 2020 PLoS One, developed a helpful graphic which you may want to adapt for restructuring the methods section. | A graphical representation of the method section using RE-AIM conceptual framework has been developed as figure 1.  Lines 161 - 165 |
|  | It would be helpful to readers unfamiliar with iCCM and IMCI to better understand these programs and how the PSBI intervention is related (or not) to them. Currently these are mentioned with references but given these are existing platforms from which the intervention is building upon, they warrant description. | The components of the implementation processes are from iCCM and IMCI guidelines. The two documents have been referenced for additional information. References 14 and 16 |
|  | Figure 1 is blurry. These results should be discussed and additional data shown by month (even as a supplementary file). Why was there an increase in the second period? | The previous figure 1 has been changed to figure 2 and formatted to make it clearer. Additional data shown in months has been included as a supplementary file S1 Figure. The increase in the second period was due to improved community awareness and acceptability of the project with increasing project duration. This has been included in lines 330 – 331 and discussed in lines 427 - 430 |
|  | Table 2 provides the results of clinical treatment failure excluding deaths. This term should be defined and explained. | Clinical treatment failure has been defined under Table 2 in lines 375 – 383 |
|  | Page 20/Table 3 presents the challenges and solutions but neglects to consistently specify the timeframe of these challenges, actions and outcomes. For example, you note that the problem on time was resolved by the third month of the project, but you do not indicate other timeframes for the other challenges identified. | Timeframe has been included in Table 3 lines 399 - 400 |
|  | Panel 2 indicates treatment would be provided at the PHC on outpatient bases. Yet Table 3 indicates that sick infants were seen at home by TSU nurses during the strike period. This should be mentioned in the limitations section. It was also a learning opportunity to see how home delivery of the service by TSU nurses on how this may or may not have impacted the results – which should be discussed. | Treatment of sick young infants by TSU nurses during strike has been included in the study limitations in lines 510 – 512. |
|  | The results should provide information on the implementation experience. For example, you have included information about how long it took to establish the program in the discussion section (page 24 lines 429-430); yet given you have included the process of establishing the project in the methodology, this information should come in the results section. | Lessons learnt have been included under the sub-heading lessons learnt and scale support for other stakeholders in lines 402 – 411 |
|  | The site for the other study, Kaduna, had a free MNCH policy whereas you have indicated that the cost of treatment is mainly out of pocket in Oyo State. Did that play a role in the comparative outcomes? Were any lessons learned regarding this? | In this project the services were rendered free. This platform was similar to that of Zaria as stated in lines 264 – 267 |
|  | How do your findings link to iCCM and IMCI implementation in Oyo State or in other contexts in Nigeria? And in the other literature? | The national iCCM and IMCI implementation guideline were considered before we arrived at the recommendation at national dialogue for this project with regards to the roles and responsibilities of CORPs, CHEWs, CHOs and nurses at the level of PHCs. See Panel 2 line 182 |
|  | Were there referral issues identified (lack of petrol for the ambulances)? | Common reasons that were identified as barriers to accepting referral advice were listed in lines 444 – 446. None of the PHCs selected for the study have ambulances. |
|  | Under study site characteristics, add mortality and related coverage indicators to give a sense of situation. | Infant and neonatal mortality, antenatal care, delivery and vaccination coverage for the state has been added under study site characteristics in lines 119 – 124 |
|  | Page 6, Line 127, please name the four wards selected. | The four wards selected have been included under study site characteristics in lines 131-132. |
|  | Page 7, Lines 146-7: What is the capacity of these referral hospitals e.g. is there oxygen? Adequate space? Family centered care (e.g. space for parents)? This information would be useful given it may be factors linked to parental decision to accept referral or not. | Additional information in the referral centres have been included in the health system structure section lines 152 - 156 |
|  | Page 11, Line 181: Provide more details on the meetings organized with the community leaders (how many and when)? | More details have been included under community sensitization in lines 194-202. |

| **Reviewer** | **Comment** | **Response** |
| --- | --- | --- |
| Reviewer 2 | Abstract: the introduction needs to have a few modifications made to ensure that it reads more easily. The English in the abstract is not of the same quality compared to the rest of the paper. | The introduction of the abstract has been modified, Lines 49-53. |
|  | 2.5 million deaths ….this is globally and should be stated. | Globally has been added under introduction section. Line 93 |
|  | starts with “neonatal” infections. However, later in the introduction it mentions about “young infants up to 2 months of age”. The definition of “neonatal” is less than 1 month of age. | PSBI management guideline include infants up to 2 months of age. Link to the guideline is in reference 10 |
|  | Methods: line 115…enabling factors…. | Facilitating factors has been changed to enabling factors. Line 115. |
|  | Panel 2: font is different size | The font size in panel 1 & 2 have been changed to 12. Lines 180 - 182 |
|  | 303…assuming 30% births were missed….what evidence is this 30% based on? | References from similar study areas have been included. Lines 331 – 332 |
|  | 304….approximately 45% cases…..what evidence supports this statement? | Obtained from home visit address register. Line 333 |
|  | Table 1: Infants referred to hospital from PHC: it is stated in column 2,3,4 that 100% of these patients were referred to the hospital. However, the text suggests that the column 2 should be 21/338, column 3 63/462 and column 4 17/22. | All sick infants were first referred to the hospital, only those that refused referral were further reclassified and treated on outpatient basis. The suggestions do not tally with the interpretation of the table 1. |
|  | 458 in conclusion…there should be a separate heading of “Conclusion” | Conclusion heading has been created. Line 514 |
